# How are medical schools supporting student’s mental wellbeing during the COVID-19 pandemic?

**DOI:** 10.1192/j.eurpsy.2022.1350

**Published:** 2022-09-01

**Authors:** R. Howard, V. Selwyn, J. Beezhold, N. Henderson, R. Gilmore, I. Bartolome

**Affiliations:** 1 University of East Anglia, Norwich Medical School, Norwich, United Kingdom; 2 University of East Anglia, Psychiatry, Norwich, United Kingdom

**Keywords:** medical student, mental wellbeing, medical school, Covid-19 pandemic

## Abstract

**Introduction:**

The COVID-19 pandemic has impacted medical students in many ways. They are not exempt from personal struggles caused by the health crisis, and many have faced similar challenges adapting to a new learning experience. The University of East Anglia (UEA) has initiatives in place to support medical students including the society Headucate UEA and the Wellbeing Champions scheme established by Norwich Medical School (NMS).

**Objectives:**

Headucate aims to improve mental wellbeing by educational online webinars and social events aimed at university students. NMS Wellbeing Champions offer support and signpost students to resources and the wider student support system at the UEA.

**Methods:**

Headucate was established in 2012 by NMS students that began running workshops at local secondary schools. Their work has expanded to include wellbeing workshops, social events for students and mental health first aid training, so members can provide peer support. Wellbeing Champions are medical student representatives responsible for completing mental health first aid training, communication between students and faculty, providing resources and signposting, creating mental health bulletin newsletters, and running socials exclusively for medical students.

**Results:**

100% of Headucate workshop attendees who completed anonymous feedback agreed that they enjoyed it and that it was useful. No feedback has been collected regarding the success of the Wellbeing Champions. This should be carried out to assess and enhance the project further.

**Conclusions:**

More data is needed to establish the success of the initiatives at NMS and their impact on medical student’s wellbeing.

**Disclosure:**

No significant relationships.

